# Using MEST-C Scores and the International Study of Kidney Disease in Children Classification to Predict Outcomes of Henoch–Schönlein Purpura Nephritis in Children

**DOI:** 10.3389/fped.2021.658845

**Published:** 2021-04-14

**Authors:** Meiqiu Wang, Ren Wang, Xu He, Pei Zhang, Qianhuining Kuang, Jun Yao, Xiang Fang, Zhuo Shi, Heyan Wu, Yingchao Peng, Zhengkun Xia, Chunlin Gao

**Affiliations:** ^1^Department of Pediatrics, Jinling Hospital, The First School of Clinical Medicine, Southern Medical University, Nanjing, China; ^2^Department of Pediatrics, Jinling Hospital, Nanjing Medical University, Nanjing, China; ^3^Department of Pediatrics, Jinling Hospital, Nanjing, China

**Keywords:** Henoch–Schönlein purpura nephritis, Oxford classification, MEST-C score, IgA vasculitis, ISKDC classification

## Abstract

**Introduction:** Henoch–Schönlein purpura nephritis (HSPN) and IgA nephropathy (IgAN) bear similarities in some aspects. The histological classification of HSPN was built on the International Study of Kidney Disease in Children (ISKDC) criteria, while IgAN was established on the 2016 Oxford classification (MEST-C scores). The purpose of this paper was to discuss the predictive value of the ISKDC classification and MEST-C scores in children with HSPN.

**Methods:** We performed a retrospective study of 877 children with HSPN in a single center between 2001 and 2019. The primary outcome was defined as chronic kidney disease—estimated glomerular filtration rate (eGFR) <90 ml/min/1.73 m^2^.

**Results:** During the follow-up period of 23.3 (10.9–47.9) months, 51 (5.8%) patients reached the primary outcome. As revealed in a Kaplan–Meier plot, segmental glomerulosclerosis (S) (*P* < 0.001) and tubular atrophy/interstitial fibrosis (T) (*P* < 0.001) significantly predict poor renal outcome. Other Oxford lesions and the ISKDC classification, however, did not show a significant difference in a worse outcome. In a multivariate Cox model adjusted for pathological and clinical factors, eGFR [hazard ratio (HR) = 2.831, 95% confidence interval (95% CI) = 1.359–5.896], S lesion (HR = 3.936, 95% CI = 2.078–7.457), and T lesion (HR = 4.002, 95% CI = 1.733–9.242) were independent risk factors for the renal outcome.

**Conclusion:** This series constitutes the largest series reported so far in the literature of such patients. According to our findings, S and T of the Oxford classification, which are ignored by the ISKDC classification, could be applied to predict the renal prognosis of children with HSPN.

## Introduction

Henoch–Schönlein purpura (HSP), also referred to as IgA vasculitis, is the most common systemic small vasculitis in childhood. It is estimated that 6 to 24 per 100,000 children under the age of 17 will develop HSP (1), which is affected by ethnic background (1). In Asia, in the age group of 4 and 6 years, the estimated annual incidence was the highest (70.3 per 100,000) (1). About 20–60% of children with HSP had renal manifestations, and 1% of these patients developed end-stage renal failure (ESRD) (2). At present, we use the International Study of Kidney Disease in Children (ISKDC) criteria for the histological classification of Henoch–Schönlein purpura nephritis (HSPN) in children, which is primarily based on the number of crescents (3), but its prognostic value remains controversial. In 2016, a working team of the International IgA Nephropathy (IgAN) Network and the Renal Pathology Society updated the 2009 Oxford classification, expanding the original four histopathologic features related to renal outcomes to five: mesangial hypercellularity (M), endocapillary proliferation (E), segmental sclerosis/adhesion (S), tubular atrophy/interstitial fibrosis (T), and cellular or fibrocellular crescents (C) (4, 5). Currently, several studies have applied the updated Oxford classification to children with HSPN for predicting renal prognosis (**Table 5**) (6–10). In this paper, we use the Oxford classification and the ISKDC classification to predict the outcomes of 877 children with HSPN.

## Materials and Methods

### Patients

The retrospective survey was conducted in Jinling Hospital in the Department of Pediatric Nephrology and Rheumatology. The inclusion criteria were as follows: (1) proteinuria, hematuria, and/or renal failure associated with purpura with/without joint or abdominal pain; (2) age at renal biopsy <18 years; (3) all patients performed a renal biopsy and were histologically confirmed to have IgA deposition; and (4) at least one clinical follow-up. Exclusion criteria were as follows: (1) lack of follow-up data; (2) <10 glomeruli on biopsy; and (3) patients with hepatitis B virus infection, lupus nephritis, Alport syndrome, and other primary or secondary glomerulonephritis should be excluded. All renal biopsies were performed with the consent of the children's parents. In total, 877 children were engaged in the study.

### Clinical and Biological Data

All biological and clinical data of patients were collected at biopsy. General clinical data were collected, including the age at the onset of purpura, the duration from the onset of purpura to renal involvement, the duration between the onset of renal involvement and renal biopsy, gender, and follow-up time. Extrarenal manifestations, renal involvement, the presence of hypertension, and treatment were recorded. Urine and blood samples were taken from the patients at biopsy for a routine test, including hematuria, 24-h urinary protein, serum creatinine, estimated glomerular filtration rate (eGFR), serum albumin, serum uric acid, hemoglobin, and cholesterol.

### Definition

The definition of primary outcome was chronic kidney disease (CKD). We defined CKD as eGFR <90 ml/min/1.73 m^2^. We calculated renal survival time from the renal biopsy to the last follow-up. eGFR was calculated with the updated Schwartz formula (11). If patients were aged over 16 years, we used the Chronic Kidney Disease Epidemiology Collaboration (CKD-EPI) equation to calculate eGFR (12). We defined hypertension as diastolic or systolic blood pressure ≥95 th percentile for age, sex, and height (13). Hematuria was defined as macroscopic hematuria or hematuria with ≥3 red blood cells/HPF 3 times within 1 week. The definition of proteinuria was 24 h proteinuria >0.4 g. Hypercholesterolemia and hypoproteinemia were defined as cholesterol >5.7 mmol/L and serum albumin <25 g/L, respectively. Hyperuricemia was defined as more than 360 mmol/L for girls and more than 420 mmol/L for boys (14). The definition of ESRD was eGFR <15 ml/min/1.72 m^2^ or requiring maintenance of renal replacement therapy for more than 3 months.

### Histological Data

Histological data (light microscopy and immunofluorescence) of the renal biopsy were recorded at the time of diagnosis. Two independent nephrologists evaluated jointly renal pathology by using the ISKDC classification (3) and the 2016 Oxford classification (MEST-C scores) (4, 5). If the two nephrologists scored differently, a third nephrologist would rescore the target and the results would depend on the third nephrologist.

### Statistical Analysis

As for the statistical methods, we used SPSS 26.0 for Windows to analyze the data. The data of normally distributed and non-normal distribution were described as mean ± standard deviation and median (interquartile range), respectively. Their comparisons were based on Student's *t*-test and the Mann–Whitney *U*-test. We used the Kruskal–Wallis test for the comparison of multiple non-normal distributions. Categorical variables were described as percentages and were compared by using the Fisher's exact test or Pearson chi-square test. Patients were divided into the following groups: ISKDC (grade I + II/III + IV + V), M0/1, E0/1, S0/1, T0/1 + 2, and C0/1/2. Their comparisons were analyzed with the Kaplan–Meier method and the log-rank test. We applied the Cox regression model to multivariate analyses. Pathological and clinical factors related to primary outcome reported in previous literature were included in the model. Variables with *P* < 0.05 in the univariate analysis were included in the multivariate analysis. These results were described as hazard ratios (HRs) with 95% confidence intervals (95% CIs). Two-sided *P* values < 0.05 were regarded as statistically significant.

## Results

### Clinical and Biological Data of Children With HSPN at Biopsy

The biological and clinical data of children with HSPN at biopsy are shown in Table 1. The median age at onset of HSP was 10.8 (8.2–13.5) years, and 60% were male. The median duration between the onset of purpura to renal involvement was 11.0 (0.0–31.0) days. The duration between the onset of renal involvement and renal biopsy was 38.0 (20.0–134.5) days. All the enrolled patients presented with renal involvement and purpura. The median follow-up time of all patients was 23.3 (10.9–47.9) months. The renal involvement was variable. Microscopic hematuria occurred in 88.6% of the patients, 16.1% of the patients had macroscopic hematuria, and 88.1% of the patients presented with proteinuria at biopsy. The median proteinuria at biopsy was 1.0 (0.6–2.0) g/day. The median hemoglobin was 130.0 (121.0–138.0) g/L. The median serum albumin and cholesterol levels were 39.2 (34.6–42.6) g/L and 4.8 (4.0–6.2) mmol/L, respectively. The median serum uric acid and serum creatinine levels were 277.0 (223.0–343.5) μmol/L and 42.8 (33.0–55.0) μmol/L, respectively. The median eGFR was 130.0 (106.6–154.1) ml/min/1.73 m^2^. All patients were divided into outcome and no-outcome groups according to renal prognosis. Compared with the no-outcome group, the duration from the onset of purpura to renal involvement was shorter, and the duration between the onset of renal involvement and renal biopsy and follow-up time were longer in the outcome group. The number of patients with hypertension in the outcome group is bigger than that in the no-outcome group. However, there were no significant differences in other clinical characteristics between the two groups.

**Table 1:** Clinical and biological data of the children with HSPN at biopsy.

	**Total (*n* = 877)**	**Outcome (*n* = 51)**	**No Outcome (*n* = 826)**	***P* value**
Male, *n* (%)	526.0 (60.0%)	36.0 (70.6%)	490.0 (59.3%)	0.111
Age at the onset of purpura, years, median, [Q1; Q3]	10.8 (8.2-13.5)	12.1 (9.1-14.0)	10.7 (8.1-13.5)	0.053
Duration between the onset of purpura and renal involvement, days, median, [Q1; Q3]	11.0 (0.0-31.0)	0.0 (0.0-21.0)	11.0 (0.0-31.0)	<0.001^**^
Duration between the onset of renal involvement and renal biopsy, days, median, [Q1; Q3]	38.0 (20.0-134.5)	94.0 (37.0-617.0)	37.5 (20.0-112.5)	<0.001^**^
Follow-up time, months, median, [Q1; Q3]	23.3 (10.9-47.9)	30.9 (16.6-55.7)	22.5 (10.7-47.1)	0.018^*^
**Renal involvement**				
Hematuria^a^, *n* (%)	781.0 (88.6%)	43.0 (84.3%)	738.0 (89.3%)	0.264
Macroscopic hematuria, *n* (%)	141.0 (16.1%)	8.0 (15.7%)	133.0 (16.1%)	0.938
Proteinuria, *n* (%)	773.0 (88.1%)	48.0 (94.1%)	101.0 (87.8%)	0.261
**Extrarenal manifestation**				
Purpura, *n* (%)	877.0 (100.0%)	51.0 (100.0%)	826.0 (100.0%)	—
Gastrointestinal symptoms, *n* (%)	367.0 (41.8%)	24.0 (47.1%)	343.0 (41.5%)	0.437
Presence of arthritis, *n* (%)	316.0 (36.0%)	17.0 (33.3%)	299.0 (36.2%)	0.648
Hypertension, *n* (%)	14.0 (1.6%)	6.0 (11.8%)	8.0 (1.0%)	<0.001^**^
**Biologic data**				
Urinary protein (g/day), median, [Q1; Q3]	1.0 (0.6-2.0)	1.0 (0.5-2.4)	1.0 (0.6-2.0)	0.776
Hemoglobin (g/L), median, [Q1; Q3]	130.0 (121.0-138.0)	130.0 (123.0-136.0)	130.0 (121.0-139.0)	0.732
Serum albumin (g/L), median, [Q1; Q3]	39.2 (34.6-42.6)	38.3 (34.7-41.5)	39.3 (34.6-42.6)	0.472
Cholesterol(mmol/L), median, [Q1; Q3]	4.8 (4.0-6.2)	4.9 (3.9-6.3)	4.8 (4.0-6.2)	0.736
Serum uric acid (μmol/L), median, [Q1; Q3]	277.0 (223.0-343.5)	268.0 (212.0-340.0)	278.0 (223.0-344.0)	0.491
Serum creatinine (μmol/L), median, [Q1; Q3]	42.8 (33.0-55.0)	48.0 (39.0-62.0)	42.1 (33.0-54.0)	0.065
eGFR (mL/min/1.73 m^2^), median, [Q1; Q3]	130.0 (106.6-154.1)	128.2 (102.8-150.5)	130.2 (106.9-154.5)	0.481
**Treatment**				
RAS blockers	349.0 (39.8%)	13.0 (25.5%)	336.0 (40.7%)	0.032^*^
*P*	790.0 (90.1%)	47.0 (92.2%)	743.0 (90.0%)	0.810
Immunosuppressive agents^b^	438.0 (49.9%)	38.0 (74.5%)	400.0 (48.4%)	<0.001^**^
MPT	321.0 (36.6%)	23.0 (45.1%)	298.0 (36.1%)	0.194

* P < 0.05;

** *P < 0.01*.

HSPN, Henoch–Schönlein purpura nephritis; Hematuria

a including macroscopic hematuria and microscopic hematuria; eGFR, estimated glomerular filtration rate; RAS blockers, Renin–angiotensin system blockers; P, prednisone; MPT, methylprednisolone pulse treatment; immunosuppressive agents

b *including mycophenolate mofetil, cyclophosphamide, calcineurin inhibitor and mizoribine*.

### Treatment of Children With HSPN After Renal Biopsy

Table 1 shows treatment after renal biopsy. In total, 790 patients (90.1%) were treated with oral prednisone and 321 patients (36.6%) received methylprednisolone pulse treatment. 349 patients (39.8%) used renin–angiotensin system (RAS) blockers and 438 patients (49.9%) received immunosuppressive agents. More frequent use of immunosuppressive agents and less use of RAS blockers were observed in the outcome group than in the no-outcome group. There was no statistical difference in using other drugs between the two groups.

### Renal Pathological Findings of Children With HSPN at Biopsy

The median number of glomerulus at renal biopsy was 24.0 (17.0–32.0). The renal pathological findings are presented in Table 2. The occurrence of immune complex deposits was as follows: 5.0% with C1q, 0.9% with C4, 71.7% with C3, 41.5% with IgM, and 26.0% with IgG. Crescents (ISKDC grade III + IV + V) were identified in 491 (56.0%) patients, including 13 (1.5%) with crescents in ≥50% of glomeruli (ISKDC grade IV + V). No crescent (ISKDC grade I + II) was identified in 386 (44.0%) patients. By using the Oxford classification, M1, E1, S1, T1/T2, and C1/C2 occurred in 38.5%, 35.1%, 30.1%, 3.0% (T1, 3.0%; T2, 0.0%), and 56.0% (C1, 46.9%; C2, 9.1%), respectively. No significant differences in M, E, and C were observed between the outcome and the no-outcome groups, except for more S and T in the former. There were differences in the composition of the ISKDC classification between the two groups.

**Table 2:** Pathological features of the children with HSPN.

	**Total (*n* = 877)**	**Outcome** **(*n* = 51)**	**No Outcome** **(*n* = 826)**	***P* value**
**ISKDC**				0.020^*^
I, *n* (%)	15.0 (1.7%)	2.0 (3.9%)	13.0 (1.6%)	
II, *n* (%)	371.0 (42.3%)	15.0 (29.4%)	356.0 (43.1%)	
IIIa, *n* (%)	441.0 (50.3%)	27.0 (52.9%)	414.0 (50.1%)	
IIIb, *n* (%)	37.0 (4.2%)	7.0 (13.7%)	30.0 (3.6%)	
IVa, *n* (%)	11.0 (1.3%)	0.0 (0.0%)	11.0 (1.3%)	
IVb, *n* (%)	1.0 (0.1%)	0.0 (0.0%)	1.0 (0.1%)	
Va, *n* (%)	0.0 (0.0%)	0.0 (0.0%)	0.0 (0.0%)	
Vb, *n* (%)	1.0 (0.1%)	0.0 (0.0%)	1.0 (0.1%)	
**MEST-C score**				
M1, *n* (%)	338.0 (38.5%)	26.0 (51.0%)	312.0 (37.8%)	0.060
E1, *n* (%)	308.0 (35.1%)	15.0 (29.4%)	293.0 (35.5%)	0.379
S1, *n* (%)	264.0 (30.1%)	34.0 (66.7%)	230.0 (27.8%)	<0.001^**^
T1 & T2, *n* (%)	26.0 (3.0%)	12.0 (23.5%)	14.0 (1.7%)	<0.001^**^
C1, *n* (%)	411.0 (46.9%)	27.0 (52.9%)	384.0 (46.5%)	0.482
C2, *n* (%)	80.0 (9.1%)	7.0 (13.7%)	73.0 (8.8%)	
**Immunofluorescence**				
IgG, *n* (%)	228.0 (26.0%)	16.0 (31.4%)	212.0 (25.7%)	0.370
IgM, *n* (%)	364.0 (41.5%)	23.0 (45.1%)	341.0 (41.3%)	0.597
C3, *n* (%)	629.0 (71.7%)	42.0 (82.4%)	587.0 (71.1%)	0.082
C4, *n* (%)	8.0 (0.9%)	2.0 (3.9%)	6.0 (0.7%)	0.074
C1q, *n* (%)	44.0 (5.0%)	4.0 (7.8%)	40.0 (4.8%)	0.316

* P < 0.05;

** *P < 0.01*.

*HSPN, Henoch–Schönlein purpura nephritis; ISKDC, the International Study of Kidney Disease in Children; a, focal; b, diffuse; M, mesangial hypercellularity; E, endocapillary proliferation; S, segmental sclerosis/adhesion; T, tubular atrophy/interstitial fibrosis; C, cellular or fibrocellular crescents*.

### Comparison of Clinical Characteristics at Renal Biopsy of Two Pathological Classifications in Children With HSPN

**Figure 1:**
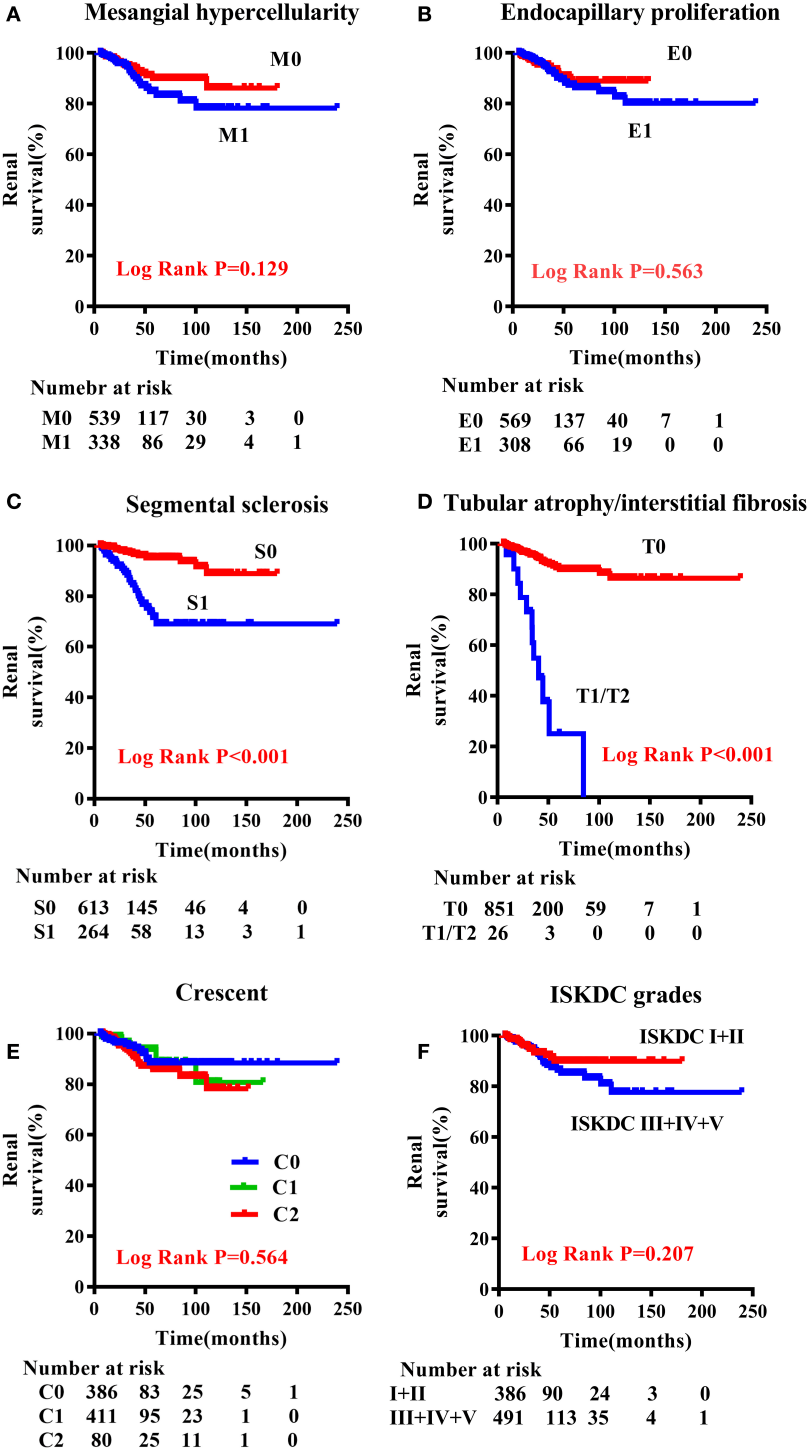
Renal outcome-free survival curves according to the Oxford classification and ISKDC classification in children with HSPN. **(A)** M0 vs. M1; **(B)** E0 vs. E1; **(C)** S0 vs. S1; **(D)** T0 vs. T1/T2; **(E)** C0 vs. C1 vs. C2; **(F)** ISKDC I + II vs. ISDKC III + IV + V. M, mesangial hypercellularity; E, endocapillary proliferation; S, segmental sclerosis/adhesion; T, tubular atrophy/interstitial fibrosis; C, cellular or fibrocellular crescents.

A comparison of the clinical characteristics at the renal biopsy of two pathological classifications in children with HSPN is summarized in Table 3. Compared with ISKDC I + II, patients had more proteinuria (*P* < 0.001), lower eGFR level (*P* < 0.001), and higher serum creatinine (Scr) level (*P* < 0.001) in ISKDC III + IV + V. The onset age of the ISKDC III + IV + V group was higher than that in the ISKDC I + II group (*P* = 0.021). Based on the updated Oxford classification, there was no significant difference in onset age between the M0 and M1 groups, but patients in the M1 group had more proteinuria (*P* < 0.001), lower eGFR level (*P* < 0.001), and higher Scr level (*P* < 0.001). There was no significant difference in Scr level, onset age, and eGFR level between the E0 and the E1 groups, but patients in the E1 group had more proteinuria than those in the E0 group (*P* < 0.001). Patients in the S1 group had a lower eGFR level (*P* = 0.026) and a higher Scr level (*P* = 0.001) than those in the S0 group. There was no significant difference in proteinuria and onset age between the S0 and the S1 groups. Patients in the T1/T2 group had a lower eGFR level (*P* < 0.001), higher Scr level (*P* < 0.001), higher proteinuria (*P* = 0.021), and higher onset age (*P* = 0.003) than those in the T0 group. Proteinuria (*P* < 0.001), Scr level (*P* < 0.001), and eGFR level (*P* < 0.001) were different among the patients in the C0 group, C1 group, and C2 group. There was no difference in onset age among the three groups. A Kaplan–Meier plot revealed that event-free renal survival was significantly shorter in patients with S1 and T1/T2 according to MEST-C scores than in those with S0 (*P* < 0.001) and T0 (*P* < 0.001) (Figure 1). However, other Oxford lesions and the ISKDC classification did not show a significant difference in event-free renal survival (Figure 1).

**Table 3:** Comparison of clinical features at renal biopsy according to two pathological classifications in the children with HSPN.

	**Onset age (years)**	***P* value**	**Proteinuria, g/24 h**	***P* value**	**Scr, μmol/L**	***P* value**	**eGFR, ml/min/1.73m^**2**^**	***P* value**
M0	10.8 (8.1-13.5)	0.998	0.8 (0.5-1.5)	<0.001^**^	41.0 (32.0-52.0)	<0.001^**^	134.7 (112.5-158.4)	<0.001^**^
M1	10.9 (8.2-13.5)		1.3 (0.7-2.9)		45.5 (35.0-58.2)		121.8 (100.5-148.7)	
E0	11.1 (8.1-13.5)	0.556	0.9 (0.5-1.7)	<0.001^**^	43.0 (32.8-57.0)	0.550	129.8 (105.4-155.9)	0.638
E1	10.3 (8.2-13.5)		1.3 (0.7-2.4)		42.0 (34.0-53.0)		130.3 (110.3-151.3)	
S0	10.7 (8.1-13.4)	0.139	0.9 (0.6-2.0)	0.135	41.0 (32.3-53.0)	0.001^**^	133.6 (108.8-155.9)	0.026^*^
S1	11.2 (8.6-13.8)		1.1 (0.6-2.0)		46.0 (35.0-59.0)		123.9 (104.7-150.8)	
T0	10.8 (8.1-13.4)	0.003^**^	1.0 (0.6-2.0)	0.021^*^	42.0 (33.0-54.0)	<0.001^**^	130.6 (107.5-154.9)	0.001^**^
T1/T2	13.9 (10.0-16.0)		1.3 (0.9-3.6)		61.0 (52.0-68.3)		105.5 (87.6-130.6)	
C0	10.4 (7.8-13.1)	0.758	0.8 (0.4-1.4)	<0.001^**^	39.0 (31.0-51.0)	<0.001^**^	137.6 (115.6-159.7)	<0.001^**^
C1	11.3 (8.5-13.7)		1.0 (0.6-2.1)		45.0 (34.0-56.0)		126.6 (104.4-151.6)	
C2	11.0 (8.3-13.9)		2.4 (1.4-5.3)		54.0 (44.0-65.0)		108.2 (88.6-134.1)	
ISKDCI/II	10.4 (7.8-13.1)	0.021^*^	0.8 (0.4-1.4)	<0.001^**^	39.0 (31.0-51.0)	<0.001^**^	137.6 (115.6-159.7)	<0.001^**^
ISKDCIII/IV/V	11.2 (8.4-13.7)		1.2 (0.7-2.4)		47.0 (35.0-58.0)		123.2 (100.6-150.5)	

* P < 0.05;

** *P < 0.01*.

*HSPN, Henoch–Schönlein purpura nephritis; M, mesangial hypercellularity; E, endocapillary proliferation; S, segmental sclerosis/adhesion; T, tubular atrophy/interstitial fibrosis; C, cellular or fibrocellular crescents; ISKDC, the International Study of Kidney Disease in Children; Scr, serum creatinine; eGFR, estimated glomerular filtration rate*.

### Univariate and Multivariate Cox Regression Analyses of Factors Associated With Renal Outcome

In Table 4, we used the univariate Cox regression analysis and found that sex (*P* = 0.047), hypertension (*P* < 0.001), eGFR (*P* < 0.001), serum albumin (*P* = 0.012), uric acid (*P* < 0.001), urinary protein (>1.0 g/day) (*P* = 0.042), S (*P* < 0.001), and T (*P* < 0.001) were risk factors for primary outcome. These factors with *P* < 0.05 were included in the multivariate model. eGFR (HR = 2.831, 95% CI = 1.359–5.896), S lesion (HR = 3.936, 95% CI = 2.078–7.457), and T lesion (HR = 4.002, 95% CI = 1.733–9.242) were independent risk factors for renal outcome.

**Table 4:** Univariate and multivariate Cox regression analyses of factors associated with renal outcome.

**Items**	**Univariate**		**Multivariate**	
	**HR (95% CI)**	***P* value**	**HR (95% CI)**	***P* value**
sex	0.543 (0.297-0.992)	*P* = 0.047^*^	—	—
arthralgia	0.925 (0.517-1.656)	*P* = 0.793	—	—
abdominal pain	1.324 (0.764-2.296)	*P* = 0.317	—	—
hypertension	11.899 (5.033-28.128)	*P* < 0.001^**^	—	—
macroscopic hematuria	0.869 (0.408-1.848)	*P* = 0.715	—	—
eGFR	4.464 (2.470-8.070)	*P* < 0.001^**^	2.831 (1.359-5.896)	*P* = 0.005^**^
hemoglobin	1.317 (0.711-2.437)	*P* = 0.381	—	—
serum albumin	2.790 (1.255-6.198)	*P* = 0.012^*^	—	—
cholesterol	1.382 (0.711-2.425)	*P* = 0.260	—	—
uric acid	3.359 (1.857-6.076)	*P* < 0.001^**^	—	—
urinary protein (0.4-1 g/d) (vs. Urinary protein <0.4 g)	1.802 (0.517-6.273)	*P* = 0.355	—	—
urinary protein(>1 g/day) (vs. urinary protein <0.4 g)	3.412 (1.048-11.111)	*P* = 0.042^*^	—	—
M1	1.525 (0.880-2.642)	*P* = 0.132	—	—
E1	0.837 (0.458-1.530)	*P* = 0.564	—	—
S1	5.056 (2.823-9.055)	*P* < 0.001^**^	3.936 (2.078-7.457)	*P* < 0.001^**^
T1/T2	11.495 (5.960-22.170)	*P* < 0.001^**^	4.002 (1.733-9.242)	*P* = 0.001^**^
C1 (vs. C0)	1.334 (0.737-2.412)	*P* = 0.341	—	—
C2 (vs. C0)	0.950 (0.352-2.560)	*P* = 0.919	—	—
ISKDC (I+II vs. III+IV+V)	1.452 (0.811-2.599)	*P* = 0.210	—	—

* P < 0.05;

** *P < 0.01*.

*M, mesangial hypercellularity; E, endocapillary proliferation; S, segmental sclerosis/adhesion; T, tubular atrophy/interstitial fibrosis; C, cellular or fibrocellular crescents; ISKDC, the International Study of Kidney Disease in Children; eGFR, estimated glomerular filtration rate, HR, hazard ratios; 95%CI, 95% confidence intervals*.

### Renal Outcomes

After 30.9 (16.6–55.7) months of follow-up, 51 patients (36 males) reached primary outcome and their eGFR level was 71.1 (42.1–82.1) ml/min/1.73 m^2^. The median 24-h proteinuria of these patients was 0.39 (0.25–1.41) g. Ten patients progressed to ESRD. After 22.5 (10.7–47.1) months of follow-up, the median 24-h proteinuria was 0.23 (0.14–0.37) g and their eGFR level was 134.1 (118.8–149.4) ml/min/1.73 m^2^ in the no-outcome group. Among them, 24-h proteinuria of 204 (23.3%) patients was more than 0.4 g.

## Discussion

The proportion of crescents is the main basis of the ISKDC classification which has been criticized as a too rough and insensitive method. Given many similarities between HSPN and IgAN in clinical features, histology, IgA immunological abnormalities, and coagulation abnormalities (15), using the Oxford classification of IgAN to predict the long-term outcomes of adults with HSPN was firstly published in Korea in 2014 (16). Xu et al. firstly applied the Oxford classification to the analysis of children with HSPN in 2017 (8), and there have been five articles concerning the Oxford classification to predict the prognosis of children with HSPN up to date (Table 5) (6–10). We analyzed retrospectively the data from 877 patients included in a Chinese single-center study, which constitutes the largest series of children reported so far in the literature of children with HSPN.

**Table 5:** Summary of studies about application of Oxford classification to predict prognosis in children with HSPN.

**References**	**Year**	**Country**	**Number of patients**	**Age**	**Primary outcome**	**Significant pathologic parameters**
Xu et al. (8)	2017	China	104	<18 years	≥ 50% reduction in initial eGFR or eGFR <90 ml/min per 1.73 m^2^	S1, T1/T2
Huang et al. (10)	2019	China	275	≥14 years	≥30% reduction in baseline eGFR in 2 years, doubling of Scr or ESRD	S1
Çakici et al. (9)	2019	Turkey	75	<18 years	either the onset of eGFR <90 ml/min/1.73 m^2^ or >50% decrease in eGFR from baseline or persistent proteinuria and/or hematuria without renal insufficiency	S1, T1/T2
Jimenez et al. (7)	2019	America	32	<18 years	Hypertension, eGFR <90 mL/min/1.73 m^2^, or proteinuria	S1
Yun et al. (6)	2020	South Korea	Children (113) Adults (100)	Children and adults	doubling of the baseline serum creatinine or development of ESRD during the follow-up period	Children: M1 and T1/T2 Adults: T1/T2
The present study	2021	China	877	<18 years	eGFR <90 mL/min/1.73 m^2^	S1, T1/T2

*HSPN, Henoch-Schönlein purpura nephritis; eGFR, estimated glomerular filtration rate; ESRD, end-stage renal disease; Scr, serum creatinine; M, mesangial hypercellularity; E, endocapillary proliferation; S, segmental sclerosis/adhesion; T, tubular atrophy/interstitial fibrosis*.

In our study, only 51 people reached the primary outcome. It may be related to the setting of endpoint events, active treatment, or age of the included subjects. It was proven by previous studies that clinical presentation and renal outcomes in patients with HSPN may be more severe in adults than in children (17), with an estimated 25 to 30% risk of developing CKD (18). However, ~1–7% of children with HSPN progress to renal failure (1). Substantial proteinuria is a significant factor for the progression to ESRD (19). In our study, 204 patients had proteinuria at the last follow-up, and they needed a longer follow-up to observe the progress of the disease.

We found that eGFR <90 ml/min/1.73 m^2^ at renal biopsy was an independent risk factor to affect the renal outcome in our study, indicating that the basic level of clinical data of patients could predict prognosis. Previous studies have shown that HSPN patients with severe clinical presentation had a poor prognosis. Coppo et al. (20) and Pillebout et al. (21) found that in adults with HSPN at presentation, renal function impairment was a negative prognostic factor. Some studies about children with HSPN did not include the role of eGFR in the multivariate regression model (7, 9), while others did not demonstrate that the decline of eGFR was a risk factor for prognosis (20).

In some researches concerning the Oxford classification to predict the prognosis of HSPN with adults (16, 22), E had an unfavorable influence on renal survival. However, most of the studies on children with HSPN (including our study) found that E was not related to prognosis (6–9, 23, 24). On one hand, it may indicate the difference in pathology and prognosis between adults and children with HSPN. On the other hand, several studies of IgAN have found that E1 was a risk factor for renal prognosis in patients who did not receive immunosuppressive therapy (4, 25). It is also important to note that some patients had already received immunosuppression before renal biopsy in our study, which may be related to immunosuppression-associated bias.

We found that T accounted for a certain proportion in the Oxford classification and the Hass classification of IgAN (4, 26), the ISN/RPS 2003 classification of lupus nephritis (27), and the modified semiquantitative classification (SQC) of HSPN (28), indicating the importance of T. T, which is ignored in the ISKDC classification of HSPN, may have important prognostic significance. Kim et al. (16) used the Oxford classification to predict prognosis in adults with HSPN and indicated that T had lower renal survival rates than those with T0, and T1/T2 (HR = 8.74; 95% CI = 1.40–54.38; *P* = 0.020) was independently concerned with reaching a primary event in a multivariate Cox model adjusted for pathologic and clinical factors. Çakici et al. (9), Xu et al. (8), Yun et al. (6), and our study concluded that T could be used to assess renal outcomes of HSPN. T1/T2 accounted for only 3.0% in our study. We hypothesize that it may be related to age. First, the onset age of T1/T2 was higher than in the T0 group in our group. In studies of adults with HSPN, the proportion of T1/T2 fluctuated between 11 and 54.1% (6, 16, 22), while that in children with HSPN was only 5.3–18.8% (6–8, 24). Second, the Oxford classification is primarily derived from the data of adult IgAN patients, and thus, it may need to be modified when evaluating pediatric HSPN. Similar to the conclusions of Xu et al. (8), Jimenez et al. (7), Huang et al. (10), and Çakici et al. (9), we used the Oxford classification to predict the prognosis of HSPN patients and found that S was a risk factor for renal outcome. S and T are chronic changes, which currently available treatments do not affect (8). Besides, these chronic changes are signs of irreversible damage and are more likely to be related to a worse prognosis (29).

The ISKDC classification is based primarily on the degree of crescent formation to predict renal prognosis. However, there is a lack of consensus regarding the value of crescents as a long-term predictor. In our study, C was not significantly related to poor renal prognosis. The point shared some similarities with those done by Kim et al. (16), Inagaki et al. (22), Huang et al. (10), and Xu et al. (8). Unlike the ISKDC classification, the Oxford classification accounts not only for acute lesions like M, E, and C but also chronic changes such as S and T. Acute lesions can guide the active use of immunosuppressive agents, while chronic lesions can predict prognosis.

This series constitutes the largest series reported so far in the literature of children with HSPN. The working group does not recommend the use of the Oxford classification in HSPN since patients were not included in the validation cohort. Our study provides sufficient and valid data to demonstrate that the application of the Oxford classification is effective in the prognosis of children with HSPN, but the study has some limitations. Firstly, the study is a single-center retrospective study. The incidence of HSPN in children is more common in Asian countries than in other countries due to ethnic background (1). Therefore, prospective and multinational cohort studies are needed. Secondly, the follow-up period is relatively short, so a small number of children reach the primary outcome. 204 patients had proteinuria by the last follow-up, and they needed a longer follow-up. Thirdly, treatment has an effect on prognosis. But due to the lack of established guidelines and changes in treatment during follow-up, we did not include treatment in the multivariate regression model. Fourth, immunosuppressive therapy before renal biopsy may also be a confounding factor. These factors need to be considered in future studies concerning the Oxford classification to predict renal outcomes in patients with HSPN.

## Conclusions

This series constitutes the largest series reported so far in the literature of such patients. According to our findings, S and T of the Oxford classification, which are ignored by the ISKDC classification, could be applied to predict renal prognosis of children with HSPN.

## Data Availability Statement

The raw data supporting the conclusions of this article will be made available by the authors, without undue reservation.

## Ethics Statement

The studies involving human participants were reviewed and approved by 2019NZGKJ-168. Written informed consent to participate in this study was provided by the participants' legal guardian/next of kin.

## Author Contributions

MW, RW, QK, JY, XF, ZS, HW, and YP conducted data acquisition and interpretation, and participated in the drafting of the manuscript. ZX, CG, and XH participated in the interpretation of the data, the evaluation of renal pathology, and the critical revision of the manuscript of important academic content. All authors participated in data acquisition, interpretation, and statistical analysis. All authors read and approved the final manuscript.

## Conflict of Interest

The authors declare that the research was conducted in the absence of any commercial or financial relationships that could be construed as a potential conflict of interest.
